# Supplementary material for: Nanoemulsion Stabilized by Safe Surfactin from Bacillus subtilis as a Multifunctional, Custom-Designed Smart Delivery System
Source: Pharmaceutics. 2020 Oct 10;12(10):953. doi: 10.3390/pharmaceutics12100953 (PMC7601209; doi:10.3390/pharmaceutics12100953)
Supplement: Supplementary file 1 [file pharmaceutics-12-00953-s001.pdf]

pharmaceutics

MDPI

Supplementary Materials: Nanoemulsion Stabilized by Safe Surfactin from *Bacillus subtilis* as a Multifunctional Custom-Designed Smart Delivery System

Agnieszka Lewińska, Marta Domżał-Kędzia, Anna Jaromin and Marcin Łukaszewicz

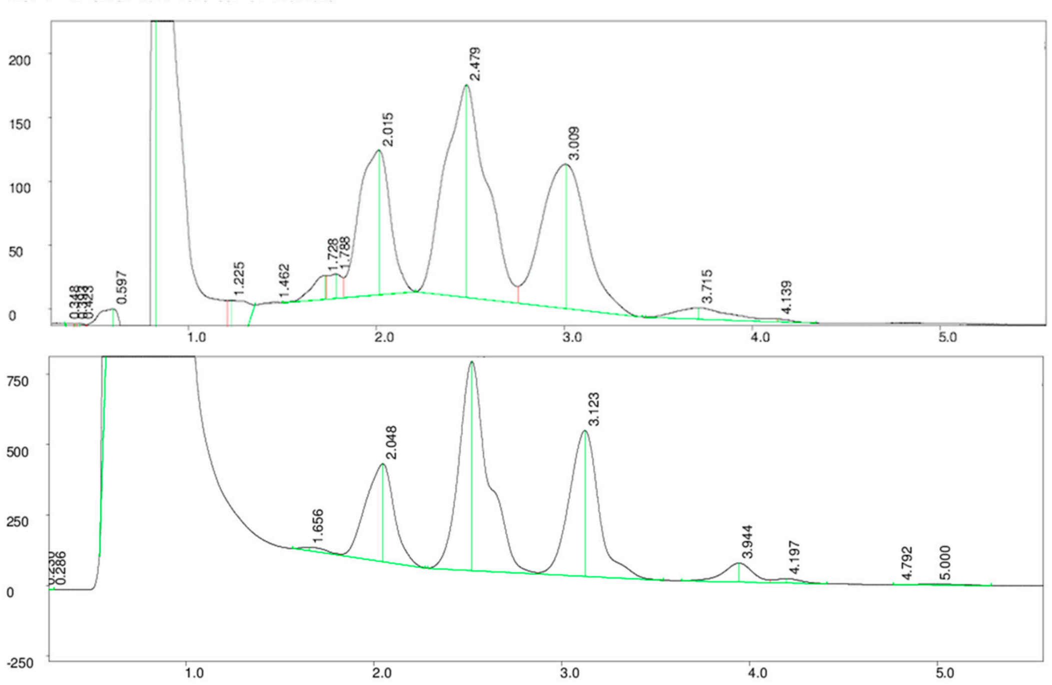

Figure S1. Analytical chromatogram of surfactin standard (up) and cultivation sample (down).

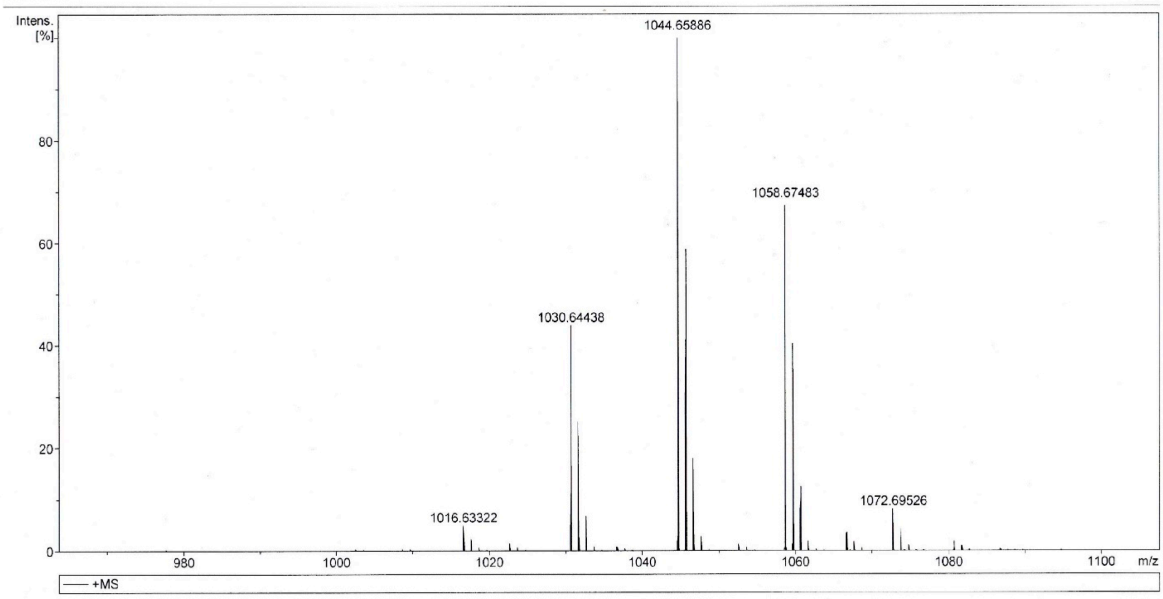

Figure S2. ESI-MS of cultivation sample. All ions are with sodium ion $[M+Na]^+$.

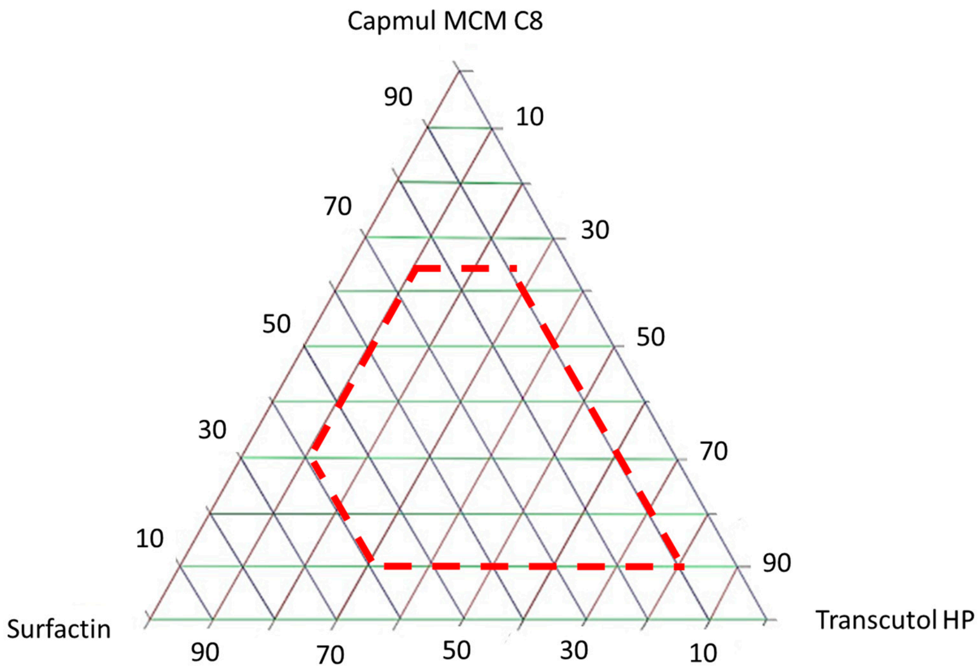

Figure S3. Phase diagram of Capmul MCM C8, surfactin and Transcutol HP.

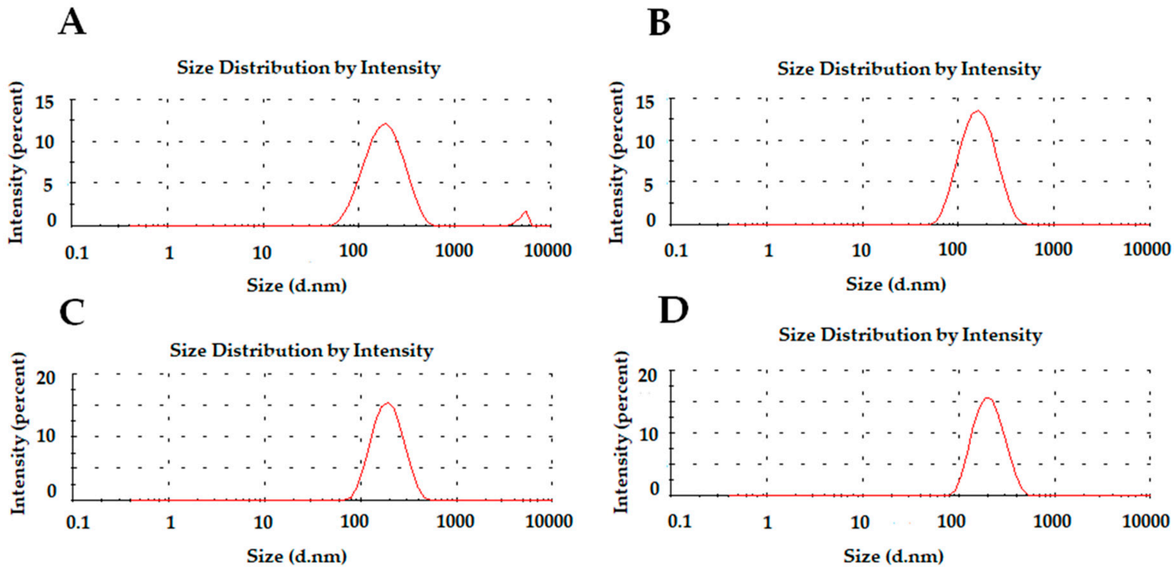

Figure S4. Size distribution of nanoemulsion with tocopherol A) after the preparation, B) after 195 days (up), nanoemulsion with ascorbyl tetraisopalmitate C) after the preparation, D) after 195 days.

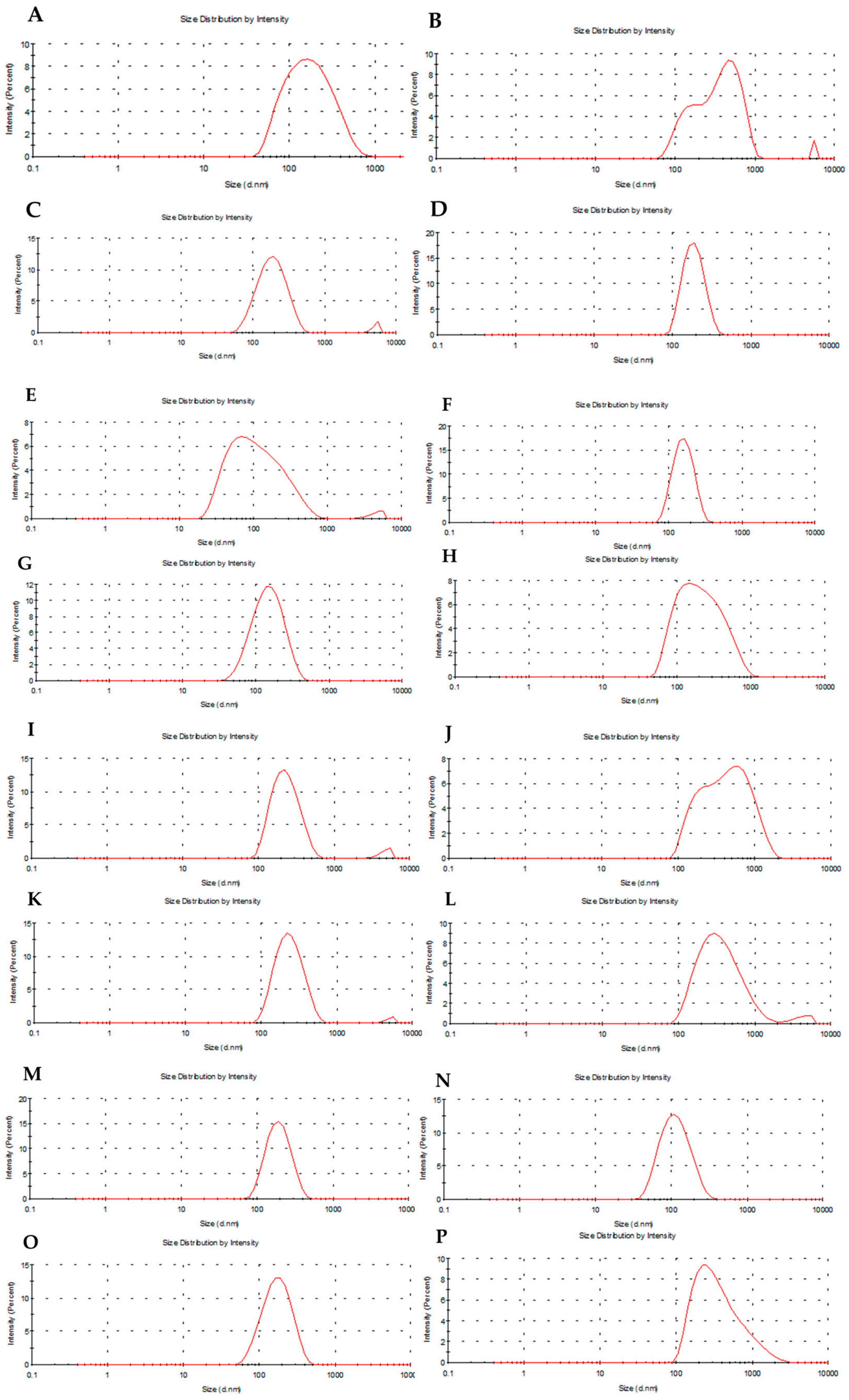

Figure S5. Size distribution of nanoemulsion with different oil phase: a) oleic acid, b) Limanthes alba (Meadowfoam) seed oil, c) Tocopherol in Helianthus annuus oil, d) Cananga oil, e) Listea Cubeba Fruit Oil, f) Buckwheat Oil organic, g) Carrot Seed oil organic, h) Chaulmoogra Oil Organic, i) tocopherol, j) Tamanu Oil pressed, k) Avocado oil pressed, l) Pomegranate Oil pressed, m) Ascorbyl

Tetraisopalmitate, n) Passilflora Incarnata seed oil, o) Rapeseed oil, p) Seabucktorn Pulp Oil, r) Fatty acid ethyl esters, s) Capryloyl glycine.

Table S1. Surfactin isoforms content in both standard and cultivation samples. Both in Area Under Curve [AUC] absolute unite, retention time in the minute.

SRF isoforms	Retention time [min]	Sample [AUC]	STD 0.4 mg/ml [AUC]
**C12**	1,656	1,99	3,4
**C13**	2,048	60,99	21,79
**C14**	2,479	134,46	42,91
**C15**	3,123	94,39	31,62
**C16**	3,944	12,06	3,14

Table S2. Molecular mass, formula and quasimolecular ions of surfactin analogs.

SRF isoforms	Formula	Molar Weight [g/mol]	$[M+Na]^+$ [g/mol]
C12	$C_{50}H_{87}N_7O_{13}$	994.26	1016.63
C13	$C_{51}H_{89}N_7O_{13}$	1008.29	1030.64
C14	$C_{52}H_{91}N_7O_{13}$	1022.32	1044.65
C15	$C_{53}H_{93}N_7O_{13}$	1036.34	1058.67
C16	$C_{54}H_{95}N_7O_{13}$	1050.37	1072.69

Table S3. DLS analysis for different oil phase in designed nanoformulation.

No	Oil	Z-Ave	PdI	ZP
		d.nm		mV
**1**	Listea Cubeba Fruit Oil	86.78 ± 4.58	0.410 ± 0.035	-56.83 ± 8.33
**2**	Tocopherol	150.26 ± 52.85	0.656 ± 0.248	-58.83 ± 3.45
**3**	Tamanu oil	482.07 ± 21.64	0.487 ± 0.019	-106.00 ± 1.00
**4**	Oleic acid *	147.97 ± 2.90	0.256 ± 0.014	-87.63 ± 2.19
**5**	Cananga oil organic *	174.23 ± 2.32	0.077 ± 0.010	-73.50 ± 1.80
**6**	Rapeseed oil	780.37 ± 117.67	0.681 ± 0.034	-103.33 ± 1.53
**7**	Fatty acid ethyl ester *	164.90 ± 2.98	0.235 ± 0.008	-93.47 ± 5.34
**8**	Capryloyl glycine	78.82 ± 2.42	0.407 ± 0.037	-85.67 ± 0.65
**9**	Limanthes alba (Meadowfoam) seed oil	536.63 ± 302.77	0.685 ± 0.277	-99.13 ± 6.17
**10**	Passilflora Incarnata seed oil	570.57 ± 7.39	0.992 ± 0.014	-63.07 ± 1.46
**11**	Pomegranate oil organic *	143.37 ± 2.24	0.236 ± 0.008	-70.87 ± 1.53
**12**	Buckwheat Oil organic *	147.30 ± 3.99	0.114 ± 0.033	-75.40 ± 3.58
**13**	Carrot Seed oil organic *	134.07 ± 1.62	0.198 ± 0.011	-85.47 ± 3.04
**14**	Chaulmoogra oil organic *	166.97 ± 3.04	0.253 ± 0.005	-82.47 ± 2.68
**15**	Avocado oil pressed *	228.33 ± 4.48	0.232 ± 0.030	-51.87 ± 1.10
**16**	Seabuckthorn Pulp Oil *	288.90 ± 4.55	0.233 ± 0.012	-73.17 ± 0.80
**17**	Olive oil	*Two phase after preparation*		
**18**	Camelina oil	*Two phase after preparation*		
**19**	Buriti oil	*Two phase after preparation*		
**20**	Argan oil pressed	*Two phase after preparation*		
**21**	Tsubaki oil	*Two phase after preparation*		
**22**	Hemp oil pressed	*Two phase after preparation*		
**23**	Vegetable oil	*Two phase after preparation*		
**24**	Apricot Kernel Oil	*Two phase after preparation*		
**25**	Sesame Oil	*Two phase after preparation*		
**26**	Opuntia Ficus –indica seed oil	*Two phase after preparation*		

** unstability after 7 days*
